# Pediatric Obesity: Complications and Current Day Management

**DOI:** 10.3390/life13071591

**Published:** 2023-07-20

**Authors:** Mary Ellen Vajravelu, Emir Tas, Silva Arslanian

**Affiliations:** 1 Center for Pediatric Research in Obesity and Metabolism, UPMC Children’s Hospital of Pittsburgh, 4401 Penn Ave., Faculty Pavilion 6th Floor, Pittsburgh, PA 15224, USA; tase2@upmc.edu (E.T.); silva.arslanian@chp.edu (S.A.); 2 Division of Pediatric Endocrinology, Diabetes, and Metabolism, University of Pittsburgh School of Medicine, Pittsburgh, PA 15213, USA

**Keywords:** obesity, pediatric, type 2 diabetes mellitus, dyslipidemia, hypertension, obstructive sleep apnea, PCOS, anti-obesity medications, bariatric surgery

## Abstract

Obesity affects approximately 1 in 5 youth globally and increases the risk of complications during adolescence and young adulthood, including type 2 diabetes, dyslipidemia, hypertension, non-alcoholic fatty liver disease, obstructive sleep apnea, and polycystic ovary syndrome. Children and adolescents with obesity frequently experience weight stigma and have an impaired quality of life, which may exacerbate weight gain. Pediatric obesity is typically defined using sex-, age-, and population-specific body mass index percentiles. Once identified, pediatric obesity should always be managed with lifestyle modification. However, adolescents with obesity may also benefit from anti-obesity medications (AOM), several of which have been approved for use in adolescents by the US Food and Drug Administration, including liraglutide, phentermine/topiramate, and semaglutide. For children with specific, rare monogenic obesity disorders, setmelanotide is available and may lead to significant weight loss. Metabolic and bariatric surgery may be used for the management of severe obesity in youth; though highly effective, it is limited to specialized centers and has had relatively low pediatric uptake. In this narrative review using pediatric-focused data from original research, reviews, clinical practice guidelines, governmental agencies, and pharmaceutical companies, we review obesity-related metabolic complications in youth and management strategies, including AOM and bariatric surgery.

## 1. Introduction

Obesity is a growing threat to children’s health globally, affecting approximately 20% of children and adolescents in the United States [1] and worldwide [2]. This change has been due largely to the increasing access to, and affordability of, ultra-processed and energy-dense foods, as well as reduced physical activity [3]. Striking inequities in obesity prevalence by race/ethnicity, and socioeconomic status exist [4,5] (though the role of income is reversed in low- versus middle- or high-income countries [6]), contributing to widening disparities in the incidence of obesity-related conditions such as youth-onset type 2 diabetes (T2D) [7,8]. The emergence of such obesity-related complications in childhood underscores the critical importance of pediatric obesity prevention and treatment, as well as assessing for complications including T2D, dyslipidemia, hypertension, non-alcoholic fatty liver disease, obstructive sleep apnea, and polycystic ovary syndrome [9]. Lifestyle management through behavioral modification has a central role in reducing the risk of obesity-associated comorbidities, though the intensity and duration of programs that drive success can also be infeasible for clinicians to implement and for youth and families to attend. In recent years, the United States (US) Food and Drug Administration (FDA) has approved several AOMs for adolescents with obesity. In 2023, the American Academy of Pediatrics (AAP) released clinical practice guidelines (CPG) endorsing the use of such therapies without delay among youth with obesity who are aged 12 and older, regardless of the presence or absence of comorbidities [9]. These guidelines also support the use of metabolic/bariatric surgery (MBS), a highly effective therapy that can improve cardiovascular risk factors, but which has had a relatively low uptake among the adolescent population despite increases in recent years [10,11].

For this narrative review, we include data relevant to the pediatric population, including that reported in original research manuscripts, reviews, and clinical practice guidelines indexed to PubMed; publications from governmental agencies, including the US Centers for Disease Control, the National Center for Biotechnology Information, the World Health Organization, and the US Food and Drug Administration; ClinicalTrials.gov; and prescribing information from pharmaceutical agencies. This review includes data primarily from 2008 to 2023 but includes earlier publications in the case of foundational references or if inadequate data were available in the preferred timeframe. Using data from these sources, we discuss the most common metabolic complications of pediatric obesity as well as management strategies, including lifestyle modification, pharmacotherapy, and MBS.

## 2. Definitions and Classification

Pediatric obesity is typically defined using body mass index (BMI), the ratio of weight (kilograms) over squared height (meters). This measure was developed to evaluate adiposity among healthy adult men [12], and its correlation with relative body fat, particularly in younger children, is weak to moderate [13]. Its predictive utility for pediatric overweight translating to adult obesity may also differ by race [14]. Although more direct measures of adiposity, such as triceps skinfold thickness, exist, BMI is often preferred due to its ease and greater inter-and intra-observer reliability in measurement [15]. Due to growth throughout childhood, pediatric obesity definitions use BMI percentiles or the degree of difference from the mean (standard deviation score, SDS) rather than absolute BMI [16]. In the US, pediatric obesity is defined as BMI ≥ 95th percentile and severe obesity as >120% of the 95th percentile (or >35 kg/m^2^) using age- and sex-specific growth charts developed by the Centers for Disease Control and Prevention [17]. Severe obesity can be further subdivided into Class 2 (BMI 120% to <140% of the 95th percentile, or BMI 35 to <40 kg/m^2^) and Class 3 Obesity (BMI ≥ 140% of the 95th percentile, or BMI ≥ 40 kg/m^2^) [9]. In contrast, the World Health Organization (WHO) uses internationally derived growth charts and defines obesity as BMI-for-age greater than two standard deviations above the WHO Growth Reference median for children ages 5–19 years and as weight-for-height greater than three standard deviations above the WHO Child Growth Standards median for children younger than 5 years [2].

## 3. Complications of Pediatric Obesity

### 3.1. Type 2 Diabetes and Prediabetes

Once considered a disease of adulthood, youth type 2 diabetes (T2D) has risen in incidence in the past two decades [7,8], with further increases during the COVID-19 pandemic [18]. Youth-onset T2D disproportionately affects people of color, including Black, Hispanic, Asian, American Indian/Alaskan Native, and Pacific Islander populations [8,19]. In the US, the prevalence of T2D in youth ages 10–19 years is nearly 10-fold higher among Black (1.8 per 1000) and American Indian (1.63 per 1000) youth and 3–5-fold higher among Hispanic and Asian youth (1.03 per 1000, 0.59 per 1000, respectively) than non-Hispanic white youth (0.2 per 1000). Although T2D may be diagnosed prior to puberty in rare cases [20,21], it is significantly more common among older adolescents (15–19 years) as compared to younger (10–14 years) [7]. T2D is also more common among female than male adolescents [7], despite the reverse sex pattern in adulthood [22]. As documented by the Treatment Options for Type 2 Diabetes in Adolescents and Youth (TODAY) study and the SEARCH for Diabetes in Youth study [23,24], individuals with youth-onset T2D face diabetes-related complications soon after diagnosis [23] and throughout early adulthood [24], with higher prevalence of microvascular complications than individuals with type 1 diabetes matched for disease duration, glycemic control, and age [25]. Obesity appears to play an important role in macrovascular complications of T2D, such as coronary heart disease, peripheral artery disease, cardiomyopathy, arrhythmias, and cerebrovascular disease: in the SEARCH study, although youth with T2D had a significantly higher prevalence of hypertension and arterial stiffness than youth with type 1 diabetes, adjustment for obesity status attenuated these differences in prevalence [25].

The rising incidence of T2D has paralleled trends in childhood obesity with a latency of approximately 10 years [26] and reflects a complex set of exposures both contributing to and stemming from obesity, including the prenatal environment, feeding practices in infancy, and environmental exposures throughout childhood [27]. Due to the risks of untreated T2D, including prolonged hyperglycemia that may increase the risk of diabetes-related complications, screening is recommended for youth at risk of T2D [9,28,29]. Among youth with BMI ≥ 85th percentile who are at least 10 years old or pubertal, the American Diabetes Association, American Academy of Pediatrics (AAP), and International Society for Pediatric and Adolescent Diabetes (ISPAD) recommend screening if at least one additional risk factor is present (Table 1) [9,28,29]. Screening can be achieved by measuring glycated hemoglobin (i.e., HbA1c), fasting serum glucose, or, less commonly, via a 2 h oral glucose tolerance test (OGTT) [30]. The most common outcome of screening is identification of prediabetes, which encompasses impaired fasting glucose (100–125 mg/dL; 5.6–6.9 mmol/L), elevated HbA1c (5.7–6.4%; 38.8–46.4 mmol/mol), and impaired glucose tolerance (2 h glucose during OGTT 140–199 mg/dL, 7.8–11.1 mmol/L). Like T2D, prediabetes has also risen in incidence in youth and is now present in nearly 30% of adolescents aged 12–18 years old [31]. It is also more common among males than females, the opposite of T2D in youth [31]. Despite the high rate of progression from prediabetes to T2D among adults, the relevance of this diagnosis is less certain in youth, as more than 50% may revert to normoglycemia after puberty [32], and mild elevations of hemoglobin A1c (5.7–5.9%) infrequently progress to T2D within two years [33].

The primary treatment approach to prediabetes in youth is lifestyle modification [29]. Although the use of metformin among adults with prediabetes has been shown to slow the progression to T2D [34], data are limited in youth and do not appear to be as favorable. As shown by the Restoring Insulin Secretion (RISE) Study, which randomized youth with impaired glucose tolerance or recently diagnosed T2D 1:1 to either metformin alone for 12 months or 3 months of insulin glargine followed by 9 months of metformin, the rate of glycemic worsening did not differ between groups and occurred in 17.8% and 36% of youth overall at 12 and 21 months, respectively [35]. This was significantly higher than an adult comparator group, in whom glycemic worsening occurred in 7.5% and 20% at 12 and 21 months, respectively [35]. Based on the findings from RISE and insufficient long-term data, the use of metformin or insulin is not recommended for youth with prediabetes [29].

### 3.2. Dyslipidemia

Dyslipidemia is a common but overlooked complication of pediatric obesity. In fact, obesity is the most common cause of secondary lipid disorders. Dyslipidemia is a major risk factor for cardiovascular disease (CVD) morbidity and mortality, and it is closely associated with insulin resistance and other components of the metabolic syndrome [36]. Dyslipidemia is associated with increased carotid intima media thickness (cIMT), a harbinger of future adverse cardiovascular events. The importance of primary prevention of dyslipidemia was demonstrated by the prospective “Cardiovascular Risk in Young Finns Study”, which found that childhood dyslipidemia, independent of obesity status, was a risk factor for carotid plaque formation and correlated with its size in adults, even if dyslipidemia was resolved by adulthood [37].

Current clinical guidelines recommend universal lipid screening between the ages of 9 to 11 years and again between 17 to 21 years [38]. Even younger children with obesity may have an abnormal lipid profile [39]. Forty percent of children with obesity are estimated to have some form of dyslipidemia [40]. Although a non-fasting lipid panel is useful to screen for hypercholesterolemia, its utility in screening for obesity-associated lipid disorders is limited because serum triglyceride levels are affected by dietary fat and carbohydrate intake. Elevated triglyceride and low HDL cholesterol are the most commonly encountered laboratory abnormalities during routine screening for obesity, but high total and LDL cholesterol levels also occur frequently [38].

Despite the high frequency of dyslipidemia in children and universal screening guidelines, screening rates are still very low [41,42,43]. Therefore, the true prevalence of dyslipidemia is likely underestimated. Moreover, significant knowledge gaps exist among primary pediatric care providers in the diagnosis and management of lipid abnormalities, which may potentially contribute to worse health outcomes in adulthood [44].

### 3.3. Hypertension

Hypertension and obesity in children are closely linked. Obesity is the major risk factor for hypertension in children, and elevated blood pressure in childhood is associated with hypertension in adulthood [45]. Whereas the prevalence is around 3% among all US children, it is estimated that up to 25% of children with obesity have hypertension [46]. Prevalence is higher with increasing age and BMI categories. Hypertension is directly related to cardiovascular abnormalities, including left ventricular hypertrophy, increased risk of high carotid intima media thickness, and left ventricular hypertrophy, though obesity was shown to be a stronger determinant of CVD outcomes than hypertension (odds ratio, 4.17 vs. 1.03) [47]. Despite the high frequency of hypertension in children with obesity, the diagnosis is usually delayed, as evidenced by the high number of children with left ventricular hypertrophy at the time of diagnosis of hypertension [48]. Furthermore, the efficacy of anti-hypertensive treatment in children with obesity is not known, given the paucity of studies.

### 3.4. Non-Alcoholic Fatty Liver Disease

Non-alcoholic fatty liver disease (NAFLD) is the leading cause of chronic liver disease in children, particularly in Hispanic males, and is strongly associated with pediatric obesity, insulin resistance and T2D, dyslipidemia, and CVD [49,50,51,52]. Around 10% of children are estimated to have some form of NAFLD in the US, while the prevalence is estimated to be approximately 40% in children with obesity, although the prevalence data varies significantly depending on the population studied and diagnostic modality used [50,53].

The spectrum of NAFLD ranges from simple steatosis to non-alcoholic steatohepatitis (NASH), cirrhosis, and liver failure [51]. While simple steatosis may resolve through intensive health behavior and lifestyle treatment, it is generally considered to be a progressive disease. Xanthakos et al., using data from children (n = 122) with biopsy-confirmed NAFLD who enrolled in the placebo groups of two clinical trials, showed that one-third had histological features of progression within 2 years despite having received standard-of-care counseling on lifestyle modification [54]. Moreover, in a recent study by Simon et al., children and young adults with biopsy-confirmed NAFLD had significantly higher rates of mortality compared to a matched general population; the absolute risk of mortality among patients with NAFLD was 6.6% (95% CI 4.0–9.2) higher [55]. Both simple steatosis and NASH are associated with a higher adjusted rate of mortality compared to controls. NASH-related cirrhosis is estimated to be the most common cause of liver transplant in adults in the near future [56].

Recently published AAP clinical practice guidelines for the evaluation and treatment of children and adolescents with obesity recommend NAFLD screening via serum alanine aminotransferase (ALT) measurement starting at 10 years of age in those with obesity, or overweight when additional risk factors, including signs of insulin resistance, prediabetes or T2D, dyslipidemia, and sleep apnea, are present [9]. Although ALT is a readily available, cheap, and minimally invasive screening test, twice the sex-specific cut-offs (≥44 IU/L for females and ≥50 IU/L for males) have low specificity to rule out NAFLD, which limits its use [50]. Conventional ultrasonography is the most commonly used imaging technique in clinical practice for the evaluation of elevated liver enzymes, as NAFLD is a diagnosis of exclusion, and definitive diagnosis requires histological examination (i.e., obtained via liver biopsy). However, ultrasonography has limitations, which include operator dependency and the inability to detect steatosis in the early stages of the disease (i.e., when <1/3 of the liver is infiltrated with fat) [50]. In the last decade, vibration-controlled transient elastography (VCTE)-based ultrasonography techniques have gained attention for the assessment of liver stiffness (i.e., fibrosis) and fatty infiltration (i.e., steatosis), but optimal thresholds for controlled attenuated parameter (CAP) scores for NAFLD diagnosis are yet to be determined. Furthermore, VCTE-CAP score and liver fat do not correlate well in children with obesity, which further limits its ability to monitor changes in liver fat over time [57].

Magnetic resonance imaging (MRI)-based techniques are alternatives to liver biopsy in NAFLD diagnosis, as they can precisely quantify liver fat and accurately rule out other etiologies, but their high cost and limited availability limit their use in the clinical settings [58]. Given relatively low adherence rates to NAFLD screening guidelines and the poor performance of currently available clinical screening tests, including serum ALT and liver ultrasonography, true prevalence, as assessed by liver biopsy or MRI-based techniques, may be higher than estimated [59]. There are no FDA-approved pharmacotherapies for NAFLD treatment.

### 3.5. Obstructive Sleep Apnea

Obstructive sleep apnea (OSA) is significantly more common in children with obesity than those with a healthy weight (45% versus 9%) [60]. Among youth with obesity, the severity of OSA, as measured by the apnea–hypopnea index (AHI), is positively associated with visceral adiposity [61]. Moreover, OSA is linked to insulin resistance [62], NAFLD [63], and hypertension [64] in children with obesity. OSA may contribute to poor cognitive outcomes; among adolescents with severe obesity, sleep fragmentation related to OSA was associated with worse performance on standardized cognitive testing, including vocabulary and psychomotor efficiency [65]. To detect OSA in children with obesity, clinicians should take a sleep history, including snoring, somnolence, inattention, headaches, and nocturnal enuresis, and refer for polysomnography if at least one of these symptoms is present [9].

### 3.6. Polycystic Ovary Syndrome

Polycystic ovary syndrome (PCOS) is a complex metabolic disorder characterized by androgen excess and ovulatory dysfunction [66]. The estimated prevalence among reproductive-age women varies, ranging from 6 to 20%, but may be as high as 88% among women with overweight/obesity [67]. Among adolescents with PCOS, 40–70% have overweight or obesity. Clinical features related to hyperandrogenism include acne, hirsutism, male-pattern alopecia, and reproductive dysfunction, including oligoamenorrhea and sub-fertility [67]. For adolescents, the most widely accepted diagnostic criteria are the National Institutes of Health (NIH) criteria: oligomenorrhea (≤8 menses a year) and clinical or biochemical evidence of hyperandrogenism [68]. PCOS should not be diagnosed in adolescents within 2 years post-menarche, as anovulatory cycles are common during this time unless there is a strong family history of PCOS. Radiographic evidence of polycystic ovaries should not be used to diagnose PCOS in adolescents, as this finding is non-specific in this age range. Other causes of menstrual irregularity should be considered before a diagnosis of PCOS is confirmed, including congenital adrenal hyperplasia, androgen-secreting tumors, exogenous steroid/androgen exposure, hyperprolactinemia, thyroid dysfunction, and Cushing syndrome [66]. In addition, menstrual irregularities, including oligomenorrhea are common among young women with obesity but without PCOS [69].

Management of PCOS in adolescents with obesity centers on lifestyle change, including increased physical activity and nutritional counseling, together with anti-androgen treatment, including combined oral contraceptive pills, metformin, spironolactone, and dermatologic management of hirsutism and acne, as reviewed elsewhere [66,70]. Improvement in symptoms can result from weight loss [70,71], and treatment should be tailored to the individual patient based on presenting symptoms and concerns.

### 3.7. Weight Stigma, Depression, and Quality of Life

The experience of weight-related stigma is common among youth and their caregivers and may contribute to psychosocial impairment, unhealthy weight control behaviors, additional weight gain, and reduced health-related quality of life [72]. Weight stigma and obesity also appear to have a bi-directional relationship in youth, with the increased stigma associated with a BMI increase in longitudinal studies [73]. In the school setting, bullying is 51% more common among children with obesity than those with normal weight [74]. Pediatric providers can help to address these adverse experiences by modeling the use of nonbiased language and behaviors, using empowering counseling techniques, and advocating for training and awareness among clinicians about weight stigma [75]. Providers should also be aware of the increased risk of depression and anxiety among youth with obesity [76], particularly depression among adolescent females [77]. Several reviews and meta-analyses have highlighted the variability in the prevalence of depression, anxiety, and other psychiatric conditions among youth with obesity, which is due in part to the heterogeneity in patient populations studied [78,79,80]. Quality of life may be lower among youth with obesity due to obesity itself or due to comorbidities such as T2D [81], PCOS [82], or obstructive sleep apnea [83]. Obesity treatment via intensive behavioral interventions may improve the quality of life [84], and the use of highly effective weight-loss medications may improve the quality of life sub-domain of physical comfort [85].

## 4. Management of Pediatric Obesity

### 4.1. Lifestyle Interventions

A cornerstone of pediatric obesity management is the adoption of healthier dietary habits, promotion of physical activity, and reduction in sedentary behavior. Although there is overwhelming evidence for using lifestyle modifications to achieve a better state of health, best practices for nutritional intervention, exercise regimen, or behavioral health approaches to reduce weight loss in children and adolescents are not known. The most recent AAP clinical practice guidelines recommend using the term “Intensive Health Behavior and Lifestyle Treatment (IHBLT)” to emphasize treatment goals, including improved weight status and elimination of comorbidities [9]. Comprehensive interventions (i.e., those that address nutrition, physical activity, and behavior aspects) with higher intensities (i.e., greater than 26 contact hours, ideally face-to-face, over 3–12 months) were shown to yield clinically significant BMI reduction compared to less intense interventions [86]. While the inclusion of parents or family units was more likely to decrease BMI in younger children compared to interventions that do not involve parents or family units (i.e., school-based, after-school, summer camp, etc.), evidence regarding parental inclusion in adolescent weight management outcomes is less clear [87,88]. Moreover, IHBTL was shown to be more effective in younger children compared to adolescents. Despite strong evidence to support the use of IHTBL in pediatric obesity management, the effectiveness of IHTBL is significantly limited when there is a lack of family engagement, particularly when the participants do not experience a measurable change in the scale because of unrealistic expectations from the programs. In fact, the attrition rates in weight management programs can be as high as 60% [89]. Moreover, weight loss is not easy for most to achieve and sustain, particularly in those with early-onset obesity [90]. However, a multitude of studies have shown that lifestyle modifications improve overall health even in the absence of significant weight reduction [91]. Therefore, regardless of the effects of IHBLT on BMI, children and adolescents with obesity should be educated and empowered to make lifestyle changes that promote physical and mental well-being.

### 4.2. Weight-Loss Pharmacotherapy

**Figure 1:**
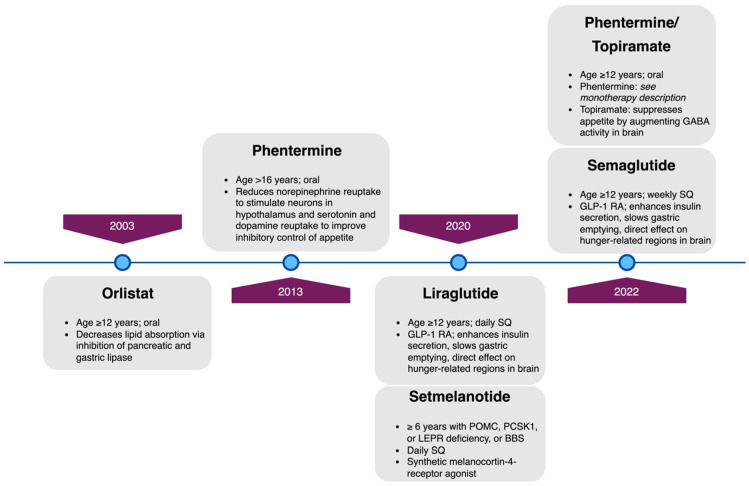
Timeline of US Food and Drug Administration approval for the use of weight-loss pharmacotherapy in pediatric patients with obesity, with minimum approved age and mechanism of action for each. GLP-1 RA, glucagon-like peptide-1 receptor agonist; POMC, pro-opiomelanocortin; PCKS1, proprotein subtilisin/kexin type 1; LEPR, leptin receptor; BBS, Bardet–Biedl Syndrome; SQ, subcutaneous.

Consistent with obesity’s status as a chronic disease with a high risk of poor long-term health outcomes [92,93], the AAP’s 2023 Clinical Practice Guidelines for the Evaluation and Treatment of Children and Adolescents With Obesity support pediatric clinicians in offering weight-loss pharmacotherapy to adolescents aged 12 and older with obesity, as defined by BMI ≥ 95th percentile for age/sex [9]. At the time of this review, four medications (Figure 1, Table 2) have been approved by the United States FDA for weight loss in youth with obesity who are ≥12 years of age: orlistat [94], liraglutide [95], phentermine-topiramate [96], and semaglutide [97]. Phentermine monotherapy is FDA-approved for patients ages > 16 years old for short-term use, limiting its applicability to younger adolescents [94,98]. One additional medication, setmelanotide [99], is FDA-approved for youth aged 6 and older with one of four rare genetic conditions leading to severe early-onset obesity: pro-opiomelanocortin (POMC) deficiency, proprotein subtilisin/kexin type 1 (PCSK1) deficiency, leptin receptor (LEPR) deficiency, and Bardet–Biedl Syndrome (BBS). However, the most frequently prescribed off-label medication for weight loss in children has been metformin [100], which does not have FDA approval for this indication and has only modest and temporary weight reduction [101]. In a meta-analysis of 38 randomized trials (n = 2199, mean age 13.7 years, daily dose range 500–3000 mg, duration 12–192 weeks), metformin reduced BMI by only 1.07 kg/m^2^ compared with controls [102]. In addition to metformin, several other non-FDA-approved medications are prescribed by some clinicians for weight loss in youth, including bupropion/naltrexone, topiramate alone, lisdexamphetamine, and other glucagon-like peptide-1 receptor agonists such as exenatide and dulaglutide, which are in the same medication class as semaglutide and liraglutide [103]. For this review, we will discuss only medications that have a US FDA-approved indication for pediatric weight loss.

### 4.3. Orlistat

Orlistat (marketed under the trade name Xenical^®^), which was approved by the US FDA in 2003 for use in children aged 12 and older, is an intestinal lipase inhibitor blocking dietary fat absorption. This mechanism often contributes to intolerable adverse effects of flatulence, fecal urgency, and steatorrhea [104]. In a multicenter randomized controlled trial of 120-mg orlistat given 3 times daily versus placebo, including 539 adolescents aged 12–16 years with obesity, all receiving behavioral therapy with mildly hypocaloric diet instructions, BMI change at 1 year favored orlistat (−0.55 kg/m^2^ versus +0.31 kg/m^2^ with placebo, *p* = 0.001). In addition, significantly more participants in the orlistat group experienced at least 5% or 10% decrease in BMI (26.5% or 13.3%, versus 15.7% or 4.5%, respectively). However, mild to moderate gastrointestinal adverse events were reported in up to 50% of the orlistat group. In a Cochrane Database systematic review of weight-loss pharmacotherapy in pediatrics, which included data from three randomized controlled trials of orlistat versus placebo, the mean difference in BMI was −0.79 (95% CI −1.08, −0.51) kg/m^2^ [101,104,112,113]. The same review summarized weight loss using data from two studies [112,113], which was moderate at −2.48 (95% CI −4.31, −0.65) kg. In clinical practice, orlistat is infrequently used, as shown by a retrospective cohort study of over 1700 children, adolescents, and young adults with obesity followed in a large unified health system between 2009 and 2018; only 23 total young adults were prescribed orlistat, and no children or adolescents [100].

### 4.4. Phentermine Monotherapy and Phentermine/Topiramate Combined Therapy

Phentermine is a central norepinephrine uptake inhibitor and nonselective inhibitor of serotonin and dopamine reuptake. It induces appetite suppression via activation of the pro-opiomelanocortin (POMC) neurons in the lateral hypothalamus [114] and is US FDA-approved for short-course therapy (12 weeks) for individuals older than 16 years, limiting its use for most adolescents [100,103]. It is available in once-daily oral doses of 7.5 mg, 15 mg, 30 mg, or 37.5 mg. Adverse effects, which are dose-dependent, include insomnia, dry mouth, tremor, headache, dizziness, mood alteration, and elevation of heart rate and blood pressure. In 2022, the US FDA approved extended-release phentermine and topiramate (Qsymia^®^) for use in chronic weight management in adolescents with obesity, aged 12 and older [96].

Topiramate is not FDA approved for weight loss, but it is FDA approved as monotherapy for seizure control (age 2 years and older) and migraine prophylaxis (age 12 years and older), leading to central appetite suppression through unknown mechanisms [115]. It may result in weight loss in some but not all, resulting in its off-label use by some providers in patients with obesity. The combined therapy of extended-release phentermine and topiramate (Qsymia^®^) was FDA approved in June 2022 for the treatment of obesity in adolescents following the demonstration that it leads to significant weight loss among adolescents aged 12–17 years with documented failure to lose sufficient weight or maintain weight loss in a lifestyle modification program [107]. In this randomized controlled trial, which evaluated placebo versus mid-dose 7.5 mg/46 mg and high-dose (15 mg/92 mg) phentermine/topiramate, BMI percent change at 56 weeks was −8.11% to −10.44% versus placebo for high-dose and mid-dose, respectively [107]. Phentermine/topiramate use improved high-density lipoprotein cholesterol and triglyceride levels, and adverse events were not more common in treatment groups than in the placebo group. Due to the potential serious risks associated with the drug, the US FDA required a Risk Evaluation and Mitigation Strategy (REMS) to manage known or potential serious risks, and the drug is dispensed only through certified pharmacies. The REMS requires counseling about the increased risk of congenital malformation in infants exposed during the first trimester, the importance of pregnancy prevention for patients of reproductive potential, and the need to discontinue Qsymia^®^ immediately if pregnancy occurs [108]. Other reported risks include suicidal ideation, insomnia, oligohidrosis and hyperthermia, cognitive slowing, slowing of linear growth, metabolic acidosis, hypokalemia, decreased renal function, kidney stones, seizures with abrupt withdrawal among individuals with a history of seizures, hypoglycemia in T2D if on insulin or insulin secretagogues, and acute angle glaucoma [116].

### 4.5. Glucagon-like Peptide-1 Receptor Agonists (GLP-1 RA): Liraglutide and Semaglutide

The weight loss effect of liraglutide and other GLP-1 RA, including semaglutide, is due to reduction in appetite and food cravings, increased satiety due in part to slowed gastric emptying, and altered food reward pathways [117]. Based on preclinical studies that demonstrated an increased risk of medullary thyroid cancer, GLP-1 RA is contraindicated in patients with personal or family history of multiple endocrine neoplasia type 2 or medullary thyroid cancer. Notably, in a recent nested case-control study using data from a French national health care insurance system, the use of GLP-1 RA by adults with T2D was associated with increased risk of all thyroid cancer (adjusted hazard ratio 1.58, 95% CI 1.27–1.95) and medullary thyroid cancer (adjusted hazard ratio 1.75, 95% CI 1.04–3.05) [118]. Similar evidence is lacking for youth due to the much more limited use to date.

Liraglutide (Saxenda^®^) was FDA approved in December 2020 for chronic weight management in adolescents aged 12 years and older with obesity. It is given as a daily subcutaneous injection with dosing initiated at 0.6 mg once daily and increased by 0.6 mg weekly as tolerated to reach a maximum dose of 3 mg daily [119]. Liraglutide at lower doses (Victoza^®^, doses 0.6–1.8 mg) has US FDA approval for the management of T2D in youth aged10 years and older [120]. As demonstrated in a randomized, placebo-controlled double-blind trial including 251 adolescents with obesity aged 12–17 years, randomized 1:1 to liraglutide 3 mg daily or placebo, liraglutide had a superior effect on BMI standard-deviation score reduction at 56 weeks [109]. The difference in BMI and weight for liraglutide versus placebo was −4.6% and −4.5 kg, respectively [109]. No substantial differences in quality of life or glycemic or cardiometabolic variables were demonstrated. The most frequent adverse events were gastrointestinal (64.8% versus 36.5% in liraglutide versus placebo groups) and led to discontinuation of liraglutide in 10 of 125 participants randomized to liraglutide [109].

In contrast to the daily frequency of liraglutide, semaglutide is given once weekly subcutaneously. Wegovy^®^ (semaglutide) was FDA approved in December 2022 as a weight loss treatment for adolescents aged 12 years old and above. In adults, subcutaneous semaglutide is approved for both T2D (Ozempic^®^, 0.5 mg, 1 mg, 2 mg) and weight loss (Wegovy^®^, 2.4 mg) but only one formulation (Wegovy^®^ 2.4 mg once weekly) is approved for pediatric use. Dosing is initiated at 0.25 once weekly for 4 weeks, then increased in 4-week intervals until the maximum dose of 2.4 mg is reached [121]. In the STEP TEENS trial, a double-blind parallel-group randomized placebo-controlled trial, 201 adolescents aged 12 to <18 years with BMI ≥ 85th percentile (overweight) and at least one weight-related comorbidity or BMI ≥ 95th percentile (obese) were randomized 2:1 to 2.4 mg subcutaneous weekly semaglutide or placebo for 68 weeks, in addition to lifestyle [85]. The primary outcome, BMI change, was significantly greater with semaglutide than placebo (mean change −16.1% with semaglutide versus +0.6% with placebo), and 73% receiving semaglutide lost 5% or more of their body weight. Moreover, semaglutide was associated with a greater proportion of participants with obesity achieving a normal weight or overweight BMI category versus those receiving a placebo (44.9% vs. 12.1%, respectively) [122]. Additionally, glycemic control (HbA1c), lipid profile, and alanine aminotransferase improved more with semaglutide than placebo, and the patient-reported outcome of physical comfort improved as well. Gastrointestinal adverse events, including cholelithiasis, were more common with semaglutide (62% versus 42%) [85]. Long-term follow-up studies on the use of semaglutide in youth are needed to determine whether the significant weight-loss effect and improved metabolic parameters translate to improved cardiovascular health outcomes in adulthood. Importantly, weight regain should be anticipated in the case of GLP-1 RA therapy discontinuation, which may occur due to supply interruption due to insurance coverage or availability, adverse effects, or patient preference.

### 4.6. Setmelanotide

Setmelanotide, available under the trade name Imcivree^®^, is a melanocortin-4 (MC4) receptor agonist, acting to restore normal function for appetite regulation among patients with disruptions upstream of the MC4 receptor [123]. Initially, it was FDA approved in November 2020 for chronic weight management in adults and pediatric patients aged 6 years of age and older with obesity due to POMC, PCSK1, and LEPR deficiency, and later in June 2022 for Bardet–Biedl syndrome (BBS), all rare genetic disorders that lead to severe early-onset obesity. Setmelanotide is given as a once-daily dose of 1–3 mg subcutaneously, and the starting dose is 1 mg for two weeks in children younger than 12 years, or 2 mg in those 12 and older. Adverse effects include skin hyperpigmentation, depression and suicidal ideation, spontaneous penile erections in males and sexual adverse reactions in females, injection site reactions, and gastrointestinal effects, including nausea and vomiting [123]. In clinical studies, which are small due to the rarity of the conditions, 8 of the 10 (80%) patients with POMC or PCSK1 deficiency and 5 of 11 (45%) patients with LEPR deficiency achieved ≥10% weight loss at 1 year [123]. Percent change in body weight from baseline to 1 year was −25.6% for patients with POMC or PCSK1 deficiency and −12.5% for patients with LEPR deficiency [110]. Among 32 patients with Bardet–Biedl Syndrome, 32.3% achieved ≥10% weight loss at 1 year [111].

### 4.7. Metabolic and Bariatric Surgery

Despite being a safe and effective adjunct therapy to lifestyle modifications for the treatment of severe obesity in children and adults, metabolic and bariatric surgery (MBS) is an underused modality, with only approximately 1% of eligible patients undergoing surgery in 2020, as estimated by the American Society for Metabolic and Bariatric Surgery (ASMBS) [124]. The American Academy of Pediatrics 2023 clinical practice guidelines endorse the ASMBS’s recommendations for considering MBS in pediatric patients ≥ 12 years of age with class 2 obesity (BMI 120% to <140% of the 95th percentile, or BMI 35 to <40 kg/m^2^) and a significant co-morbidity (including T2D, NASH, hypertension, dyslipidemia, OSA, or idiopathic intracranial hypertension), or class 3 obesity (BMI ≥ 140% of the 95th percentile, or BMI ≥ 40 kg/m^2^) even in the absence of a comorbid condition [9]. Management of pediatric obesity, in general, is enhanced by a multidisciplinary approach, including the primary care provider, registered dietitian, psychologist, and social worker. This multidisciplinary management, with the addition of surgeons, is particularly important when assessing patient eligibility for MBS, which should take an approach that emphasizes shared decision-making between patients, caregivers, and medical and surgical teams [9].

Vertical sleeve gastrectomy is the most commonly used technique in children, followed by laparoscopic Roux-en-Y gastric bypass (RYGB) [125]. Although these procedures lead to sustained weight loss (i.e., BMI reduction of 10–17 kg/m^2^ after 3 to 5 years) and cardiometabolic benefits (i.e., resolution of T2D, dyslipidemia, etc.), a considerable number of patients were reported to have experienced complications, including re-operation, gall-bladder stones, and micronutrient deficiencies, highlighting the importance of short- and long-term monitoring [126,127,128]. Moreover, Inge et al. showed that most patients (64%) who underwent RYGB, despite achieving significant reductions in BMI, still had BMI ≥ 35 kg/m^2^ [129]. In addition, a significant number of patients (up to 20–30%) were reported to regain or not achieve the desired weight loss, necessitating revision surgery [130,131]. A multitude of adult studies have reported the beneficial effects of pharmacotherapy (GLP-1 RA) in achieving further weight loss in such patients [132,133], but pediatric data assessing the efficacy of pharmacotherapy following MBS is lacking. Well-designed pediatric studies to assess and compare the effects of lifestyle interventions versus pharmacotherapy in patients who failed MBS are necessary to provide evidence-based guidance for youth.

## 5. Conclusions

Childhood obesity is an increasingly prevalent chronic disease that has negative short and long-term consequences on metabolic, physical, and psychosocial health. Obesity in children strongly predicts adult weight status and future health outcomes. Therefore, timely diagnosis and management of pediatric obesity and its complications are paramount. As reviewed above, common complications of pediatric obesity include T2D or prediabetes, dyslipidemia, hypertension, NAFLD, OSA, PCOS, and mental health concerns. Screening for these complications is sometimes limited by testing burden, cost, or invasiveness, particularly for NAFLD. Management is often limited by a paucity of approved therapies in pediatrics, as well as a lack of large-scale or long-term studies in pediatric populations. In addition to the physical comorbidities of obesity, mental health should be evaluated and addressed, with attention to disordered eating as well as experiences of bullying and obesity-related stigma that may contribute to anxiety and depression in youth with obesity.

A multifaceted patient/caregiver-centered approach promoting healthier lifestyle habits has long been the cornerstone of obesity management in pediatric patients. In the past few years, we have witnessed tremendous advances in weight-loss pharmacotherapies, which has opened a new era in pediatric obesity management, enabling clinicians to offer and discuss alternative treatment options, supplementing but not replacing Intensive Health Behavior and Lifestyle Treatment as the foundation in chronic weight management. Future studies should evaluate the real-world uptake of emerging weight-loss pharmacotherapies in adolescents, as well as the impact on metabolic health and potential solutions to prevent weight regain when the drug is discontinued for whatever reason. As prevention of adult obesity and associated complications often begins in childhood and adolescence, clinicians caring for youth have a central role in ensuring their patients are aware of and have access to lifestyle interventions, pharmacotherapy, and surgery, as appropriate. Lastly, despite the remarkable advances in treating obesity across the lifespan, prevention of obesity on a societal level remains the key to success.

## Figures and Tables

**Table 1:** Risk factors for type 2 diabetes in youth, in addition to overweight/obesity and puberty.

	ADA	AAP	ISPAD
Maternal history of diabetes or gestational diabetes during child’s gestation	x	x	x
Family history of type 2 diabetes in 1st or 2nd-degree relative	x	x	x
Race/ethnicity (Native American, African American, Latino, Asian American, Pacific Islander)	x		x
Signs of insulin resistance or associated conditions (acanthosis nigricans, hypertension, dyslipidemia, PCOS, small-for-gestational-age birth weight)	x	x	x
Use of obesogenic psychotropic medications		x	x

ADA, American Diabetes Association; AAP, American Academy of Pediatrics; ISPAD, International Society for Pediatric and Adolescent Diabetes.

**Table 2:** Weight-loss pharmacotherapy with current US Food and Drug Administration approval for use in adolescents with obesity.

Generic (Trade) Name	Mechanism	Age	Dose	Weight-Related Efficacy	Comorbidity Impact	Adverse Effects
Orlistat (Xenical^®^)	Inhibition of pancreatic and gastric lipase	≥12 years	120 mg three times daily by mouth with meals	Wt: −2.5 kg (−4.3, −0.7); BMI: −0.79 kg/m^2^ (−1.08, −0.51) [101]	FG, HDL, LDL, TG: NS; SBP: NS, DBP: −0.51 vs. +1.3 vs. placebo; ALT: NR [104]	Abdominal pain, steatorrhea, flatus, fecal urgency and incontinence, and fat-soluble vitamin deficiency
Phentermine alone (Adipex-P^®^, Lomaira™)	Reduces norepinephrine reuptake to stimulate neurons in hypothalamus	>16 years	15 mg, 30 mg, or 37.5 mg daily, oral	Wt: −3.6 kg (range −0.6 to −6.0) [105] (review, adult studies)	HbA1c, HDL, LDL, TG, SBP, DBP: NS, ALT: NR [106] (observational study, adults)	Insomnia, dry mouth, tremor, headache, dizziness, mood alteration, heart rate and blood pressure elevation
Phentermine/topiramate extended-release (Qsymia^®^)	Topiramate: suppresses appetite by augmenting GABA activity in brain	≥12 years	7.5 mg/46 mg or 15 mg/92 mg	% change in BMI at 56 weeks vs. placebo: −8.11% (−11.92, −4.31%) and −10.44% (−13.89, −6.99%) for top and mid-doses [107]	HbA1c: NS, HDL: +10% and +9% for mid- and top-dose; LDL: NS; TG: −21% for mid- and top-dose; SBP, DBP: NS; ALT: NR [107]	Topiramate: reversible cognitive dysfunction, paresthesia, metabolic acidosis; teratogenic (orofacial defects) and may decrease oral Contraceptive efficacy; counseling, serial pregnancy testing recommended [108]
Liraglutide (Saxenda^®^)	GLP-1 RA	≥12 years	3.0 mg subcutaneous once daily	BMI SDS at 56 weeks vs. placebo: −0.22 (−0.37, −0.08); relative: −4.64% (−7.14, −2.14); BMI reduction of ≥5% 43.3% vs. 18.7% [109]	HbA1c: NS; HDL, LDL, TG: NS; SBP, DBP: NS; ALT: NR [109]	Nausea, vomiting, diarrhea; increased heart rate from baseline; hypoglycemia in adolescents without type 2 diabetes (15%)
Semaglutide (Wegovy^®^)	GLP-1 RA	≥12 years	2.4 mg subcutaneous once weekly	Mean change in BMI from baseline to week 68 vs. placebo: −16.7% (−20.3, −13.2%); Weight reduction of ≥5% 73% vs. 18% [85]	HbA1c: −0.3%; HDL: NS, LDL: −7.0 mg/dL, TG: −30.2 mg/dL; SBP, DBP: NS; ALT: −14.1 IU/mL [85]	Nausea, vomiting, diarrhea
Setmelanotide (Imcivree^®^)	Melanocortin-4-receptor agonist	≥6 years with BBS or POMC, PCSK1, LEPR deficiency	3 mg subcutaneous once daily	POMC, LEPR: ≥10% weight loss in 80% of POMC and 45% of LEPR participants [110]	% change from baseline for (1) POMC or (2) LEPR (Clement): FG: (1) −17.2%, (2) NS; HDL: (1) +45.0%, (2) +19.6%; LDL: (1) NS, (2) −10.0%; TG: (1) −36.6%, (2) NS; SBP, DBP: (1) NS, (2) NS; ALT: NR [110]	New/worsened depression or suicidal ideation; sexual adverse reactions; skin hyperpigmentation (most common); injection site reaction; nausea, vomiting
				BBS: 7.9% average BMI loss [111]	% change from baseline: FG, HbA1c: NR; HDL: +4.3%; LDL: −8.8%; TG: −10.7%; SBP, DBP: NR; ALT: NR [111]	

Numbers in parentheses denote 95% CI unless otherwise indicated. NS, non-significant; NR: not reported. FG, fasting glucose; HbA1c, hemoglobin A1c; HDL, high-density lipoprotein; LDL, low-density lipoprotein; TG, triglycerides; SBP and DBP, systolic and diastolic blood pressure; ALT, alanine aminotransferase. GLP-1 RA, glucagon-like peptide-1 receptor agonist. BBS, Bardet–Biedl Syndrome.

## Data Availability

Not applicable.
